# Living labs as catalysts for integrated primary care: insights from a comprehensive community survey and co-creative design of community-based prevention and primary care

**DOI:** 10.1186/s12875-026-03467-3

**Published:** 2026-07-08

**Authors:** Nele A. J. De Witte, Kim Helsen, Sascha Vermeylen, Hilde Vandenhoudt, Ingrid Adriaensen, Lieven Janssens, Vicky Van der Auwera

**Affiliations:** 1 Centre of Expertise Care and Well-being – Living and Care lab LiCalab, Thomas More University of Applied Sciences, Geel, 2024 Belgium; 2 Politics & Public Governance, Faculty of Social Sciences, University of Antwerp, Antwerp, 2000 Belgium; 3 Centre of Expertise for Societal Public Administration, Faculty of Social Sciences, University of Antwerp, Antwerp, 2000 Belgium

**Keywords:** Living lab, Co-creation, Health literacy, Primary care, Health prevention, Citizen participation, Integrated care, Local government

## Abstract

**Background:**

Societal changes in terms of healthcare needs call for adaptations in the organisation of primary care. Policies and services need to be aligned to communities’ needs and a shift towards inclusive integrated care is warranted. However, stakeholder consultation is often limited and does not sufficiently take health literacy into account. To design integrated primary care, we need to gain insight into the characteristics and needs of communities regarding health, health literacy, and health service requirements. Additionally, we need to go beyond assessment and involve relevant stakeholders (including residents in a situation of vulnerability) in co-designing future-oriented and innovative primary care services and prevention activities.

**Methods:**

The current project consists of two studies. First, a large cross-sectional survey study was conducted to gain insight into health, health literacy, and preferences for primary healthcare and prevention in a Belgian community. The second study describes an extensive collaboration between a living lab and a local government to design multidisciplinary integrated care services in the community and includes co-creations and further survey data collection.

**Results:**

A total of 1078 residents participated in the first study. Perceived health, loneliness and demographic variable were interrelated. Health literacy was associated with age, education, perceived general health, perceived mental health, and loneliness. Participants indicated a need for multidisciplinary healthcare services and endorsed involvement of community policymakers. The multi-step process of the second study, including residents, vulnerable citizens and health professionals, led to tailored insights regarding local strategic actions to promote high-quality future-proof integrated primary care.

**Conclusions:**

Targeted recruitment for a community survey and co-creation sessions allowed for the inclusion of a large and rich sample to inform on local healthcare needs and define priorities for future-oriented health care initiatives to the local government. Community participation guided the design and implementation of structurally embedded primary care services and prevention activities. Researchers and policymakers from the wider community can use study processes and results as inspiration for inclusive healthcare redesign.

**Supplementary Information:**

The online version contains supplementary material available at https://doi.org/10.1186/s12875-026-03467-3.

## Background

The World Health Organization asserts that optimal care extends beyond formal healthcare services to encompass self-care and community health services, adopting a comprehensive approach that also includes mental health and social services [1]. Transitioning towards more integrated models of care and investing in prevention have been identified as important factors for healthcare efficiency [2]. The philosophy of integrated primary care hinges on a social model of health where equity, a broad inter-sectorial collaboration in dealing with community problems, and political support are key [3]. The relevance of healthcare reformation is further emphasized by societal changes leading to increased pressure on the existing healthcare system, such as projections indicating that an aging population is driving increased healthcare demands and constraints in healthcare workforce availability in Europe [4]. The local government has been put forwards as the key actor for facilitating primary care which is accessible, inclusive, and operates from a person-centred ‘one health’ perspective [5, 6].

Regional healthcare systems must be enhanced to continuously meet the evolving needs of the population in an inclusive manner. The European Union indicates that adapting healthcare policies and the workforce to future demand requires demographic insights, knowledge of local realities and active involvement of a wide range of stakeholders [4]. This suggest that it is important to investigate the health and healthcare needs of local communities. However, instead of being passive recipients, citizens can also actively participate in designing integrated health, care and community services, hereby improving municipalities’ accountability and aligning policies and services to communities’ needs [7, 8]. Local communities and living labs can facilitate inclusive and large-scale stakeholder consultation for healthcare reorganisation. Living labs are open innovation ecosystems with expertise in the inclusion of end-user populations and the execution of large-scale data collection in the context of exploration and co-creation of innovations [9, 10].

A key factor to reduce health inequality consists of addressing health literacy [11]. Health literacy can be defined as “people’s knowledge, motivation and competencies to access, understand, appraise, and apply health information in order to make judgments and take decisions in everyday life concerning healthcare, disease prevention and health promotion to maintain or improve quality of life during the life course” [12]. Lower health literacy has been associated with poorer health, unhealthy behaviours, and less use of preventive services [13–17]. Studies indicate that promoting health literacy not only reduces healthcare costs [18], but has health benefits on both an individual level and a societal level [19]. While it is important to be aware of the health literacy of the target population and to design policies and interventions that also serve vulnerable populations, research and participatory approaches often involve the ‘stronger and more active citizens’ while overlooking more vulnerable residents [20].

### Aims and scope

Recent policies highlight that adequate primary care should be organised according to community and stakeholder needs. Such a redesign requires insight into the health literacy, preferences and expectations of the community and it benefits from a participatory approach. A survey study aimed to engage a sample that reflected the local diversity to provide insight into population health, health literacy and its predictors as well as user needs for primary healthcare and preventive initiatives. Additionally, the current work also builds on these findings by supporting community actors in redesigning healthcare in a participatory way. This paper shares the set-up and results of several participatory community activities contributing to healthcare reorganisation, with the aim of sharing good practices and inspiring future work in this field. Before detailing the research questions and approaches, the local context will be briefly described.

The current research concerns a collaboration between a living lab and a local government with the goal of gaining new insights and shaping future integrated primary healthcare in the community. The research was conducted by LiCalab, a Belgian living lab with over 10 years of experience in supporting businesses, organizations, and local governments by including end users (citizens, care professionals, and other stakeholders) in an innovation development process. This living lab supports innovations in the areas health and care and collaborates with a broad network of local, regional, and EU partners with expertise in healthcare and well-being, including local governments. The living lab explores and validates new services and systems through activities such as co-creation, concept development, market insight, product development, piloting, and validation services. LiCalab is run by a multidisciplinary team, including researchers, panel managers, project managers, and a business developer, and it is an accredited member of the European Network of Living Labs (ENoLL).

The research activities are conducted in Belgium which has a healthcare system that covers many services and is available to almost the entire population. Policy making occurs at three levels in Belgium: the federal authority, six federated entities, and local authorities (10 provinces and over 550 municipalities). The responsibilities of healthcare planning, organisation and prevention are situated mostly at the level of the federal authority and federated entities [21]. Healthcare responsibilities of the provinces and municipalities are limited to matters of local interests and they act under the supervision of the regions. Municipalities are responsible for managing public hospitals or healthcare settings they have created. The data collection took place in a Flemish municipality (Vorselaar) where healthcare provision was under severe pressure due to a retirement wave in healthcare professionals, like general practitioners (GPs) and dentists. Residents were forced to look for alternatives in the wider region or postpone essential care. Therefore, the municipality wanted to support an integrated view on primary health in the community, putting the focus on prevention and close cooperation between different actors in the health and care sector. This included setting up a primary care centre, defined by the Belgian government as a centre which houses several individual healthcare providers (e.g., GPs, nurses, physiotherapists), who work in a multidisciplinary fashion to meet patient’s needs for qualitative, accessible, and continuous primary care (including prevention) and who are part of a “primary care” network [22].

**Fig. 1:**
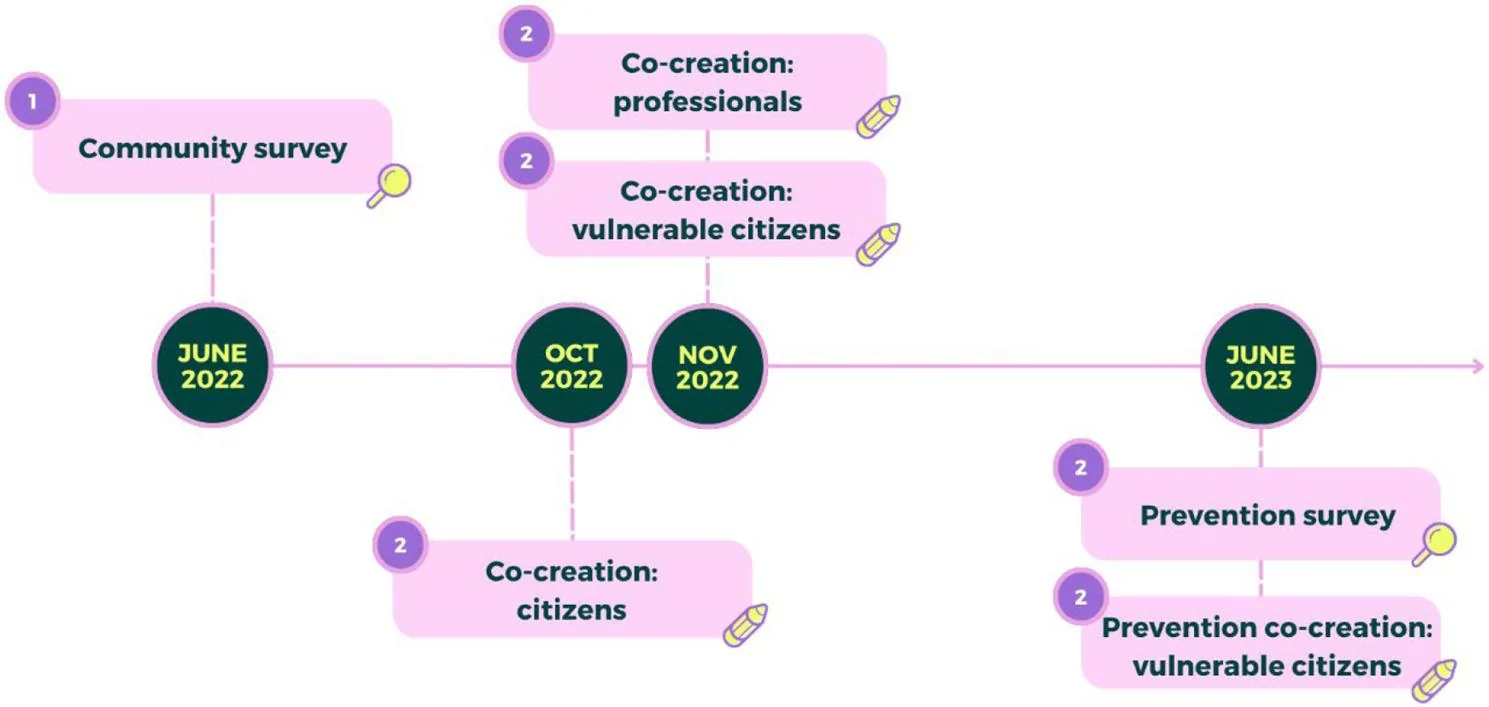
Overview of research components of the current study. Study 1 concerns the large community survey in 2022. The later stakeholder activities are described in study 2

The current research consists of two studies which contribute to new insights into user characteristics and perspectives relevant for primary care delivery. Figure 1 provides an overview of the research activities. The first study focused on the following research questions: (1) What is the health and health literacy profile of an adequate cross-section (including diversity in age, gender, education, and subjective income) of a municipality’s population, (2) which demographic factors predict health literacy, and (3) what do community members view as requirements for primary care and prevention. The second study focused on further stakeholder consultation and involvement, and how these activities help in collaboratively reorganizing primary care services within the community. This study concerns all activities performed after June 2022 (Fig. 1) and included the collection of more in-depth information from citizens, care professionals and citizens in a situation of vulnerability (e.g., citizens with lower socio-economic status) to formulate concrete and tangible goals for the (re)organisation of healthcare and prevention and gain insight into barriers and facilitators for taking part in preventive initiatives. 

## Study 1: community survey

A survey study aimed to engage a sample that reflected the local diversity to provide insight into population health, health literacy and its predictors as well as user needs for primary healthcare and preventive initiatives.

### Method

#### Recruitment

All residents of the local community (Vorselaar, Belgium) aged 18 years and above, received a personal invitation letter signed by the mayor to participate in a survey. Citizens were encouraged to complete the survey online, using a QR code or website link. Reminders to complete the survey were provided on paper through the municipal bulletin, online on the website and social media profiles of the municipality, and in person through flyers distributed at the annual fair by the panel managers of the living lab. Special attention was given to reducing participation barriers and including more vulnerable residents by e.g., by including paper questionnaires with the personal invitations sent to every adult inhabitant and by providing support in completing the online or pen-and-paper survey at different locations (such as the town hall, local service centre or even at home). Recruitment and data collection took place during 2 months in the summer of 2022. The study was approved by the Social and Societal Ethics Committee SMEC of KU Leuven (G-2022 06 2111) and all participants provided informed consent.

#### Survey

The survey consisted of four themes in addition to the demographical information (age, gender, education, professional situation, household size, knowledge of Dutch national language, and subjective poverty), perceived health and well-being (including general health, mental health, and loneliness), health literacy, needs and preferences related to both primary healthcare practice, and prevention (and the role the local government can play in this respect). The survey was provided in Dutch and included both existing (previously validated) scales and novel survey items, when no suitable existing instruments could be identified. The 6-item De Jong Gierveld loneliness scale calculates a score between 0 and 6 with higher scores representing more loneliness [23]. The scale has good internal consistency in the current sample, *α* = 0.84. The Dutch translation of the short version of the European Health Literacy Questionnaire (HLS-Q12) allows to calculate a total score ranging from 12 to 48, with higher values representing better health literacy [24]. This 12-item questionnaire has good reliability (*α* = 0.89) and is conceptually conceived as a matrix of four cognitive domains (access, understand, appraise, and apply health information) and three health domains (health care, disease prevention, and health promotion). Additionally, ranking questions, Likert scales and open-ended questions were included. For example, perceived income was defined on a 5-point Likert reflecting the extent to which household income could cover all expenses, ranging from very easy to very difficult. A translated version of the full questionnaire can be found in Supplementary file 1.

#### Data analysis

Data were analysed using SPSS 28.0 [25]. Associations between different variables were calculated using Kendall tau (t) correlations. This includes exploratory analyses regarding interrelationships between demographic variables and health status and needs. (explorative)? Hierarchical regression analyses were used to assess to which extent demographic and health variables predicted health literacy. Categorical variables were dichotomised and differences in health literacy across demographic or health factors were examined using independent t-tests. All variables showing significant t-tests were entered in the hierarchical regression analyses. Ranking questions were analysed using Friedman ANOVA. Open-ended questions were analysed through thematic analyses by KH in consultation with SV. Sample sizes can differ between analyses due to missing values.

### Results

#### Demographics

A total of 1078 inhabitants of the municipality completed the survey, of which 728 did so online. This corresponds to a response rate of 16.5%. When comparing the sample (Table 1) to population data from the Belgian bureau of statistics (Statbel; [26]), the study sample largely matched the diversity within the community. Sample characteristics showed a good distribution in age (from 18 up to 80+), employment status, and income. Nevertheless, compared to Statbel, the sample included slightly more women (60.6% as compared to 50%), slightly more individuals aged 60–79 years old (39.8% as compared to 29.8%), and fairly high education levels (33.8% non-university higher education as compared to 22.6%). One in five of the surveyed residents received little formal education. The sample had very good to native language level knowledge of the official Dutch language. The majority of respondents could easily cover household expenses with their family income while almost one in ten reported financial difficulties.

**Table 1:** Demographics

	Frequency	Percentage
Gender	1069	
Male	414	38.73%
Female	648	60.62%
X	3	0.28%
Prefer not to say	4	0.37%
Age Category	1027	
18–39 years old	220	21.42%
40–59 years old	310	30.18%
60–79 years old	409	39.82%
> 80 years old	88	8.57%
Highest education degree	1061	
No degree	43	4.05%
Primary education	68	6.41%
Lower secondary education	113	10.65%
Higher secondary education	302	28.46%
Non-university higher education	359	33.84%
University education	151	14.23%
Other	25	2.36%
Knowlegde of national Dutch language	1069	
Mother tongue	860	80.45%
Very good	113	10.57%
Good	88	8.23%
Rather limited	8	0.75%
No knowledge	0	0%
Employment status	1065	
Student	27	2.54%
Working fulltime	370	34.74%
Working parttime	142	13.33%
Not employed - looking for work	12	1.13%
Not employed - not looking for work	27	2.54%
Not employed - long-term illness or permanently incapacitated for work	28	2.63%
Retired	459	43.10%
Number of individuals in the household	1068	
1	124	11.61%
2	502	47.00%
3	168	15.73%
4	177	16.57%
5	54	5.06%
> 5	43	4.03%
Getting by on family income *(subjective income)*	1052	
Very easy	165	15.68%
Easy	389	36.98%
Neither easy nor difficult	403	38.31%
Difficult	79	7.51%
Very difficult	16	1.52%

#### Health and health literacy profile of the municipality’s population

**Fig. 2:**
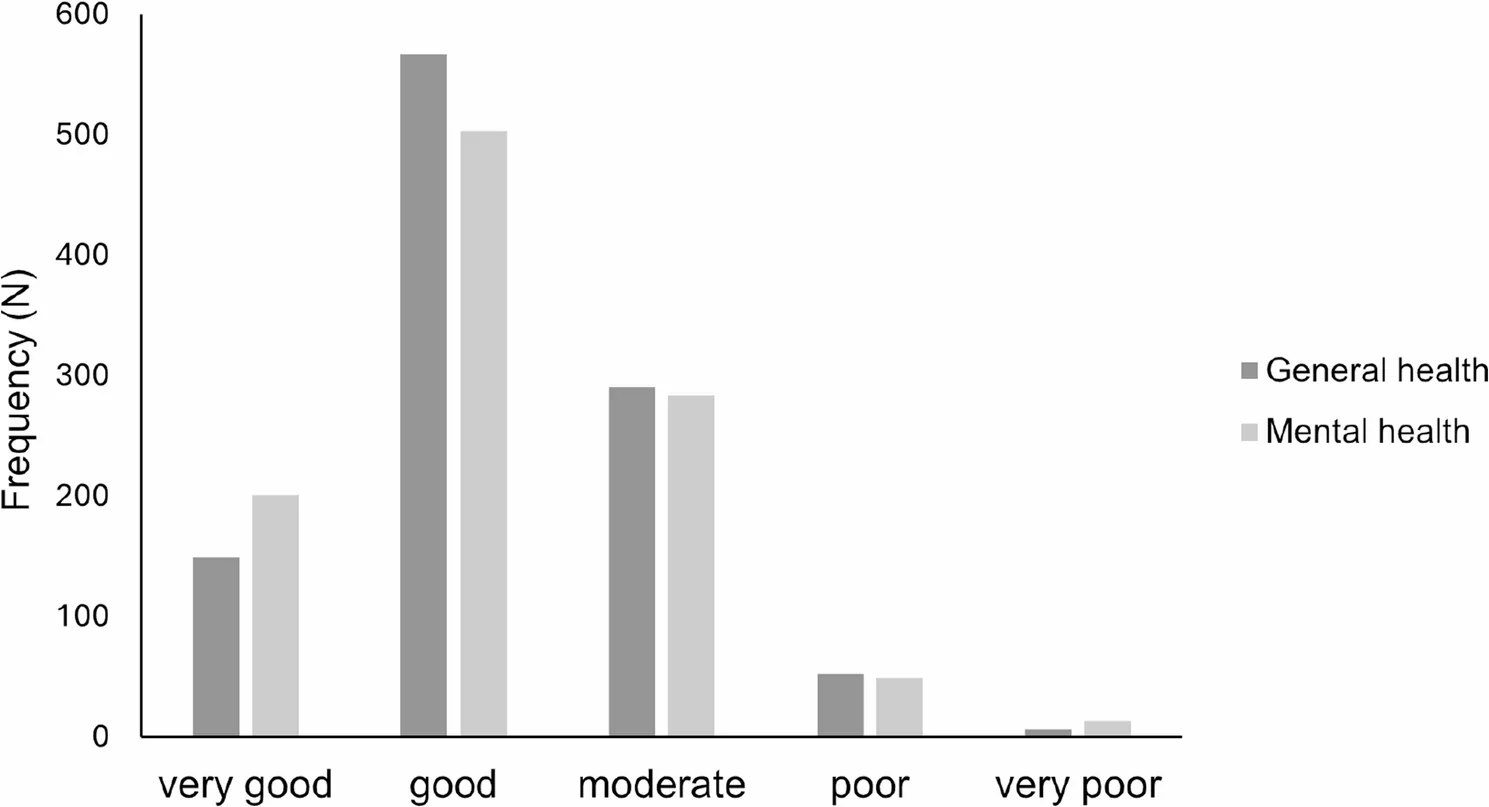
Perceived general (*N* = 1064) and mental health (*N* = 1049)

On average, respondents reported satisfactory general and mental health (Fig. 2). A minority stated to be in (very) poor general (*n* = 58/1064) or mental health (*n* = 62/1049). Feelings of loneliness were present to some extent in 53.2% of the respondents (*n* = 544/1023), with about 7.0% indicating the maximum score (*n* = 72/1023). Table 2 shows that lower general and mental health were related to lower income, lower levels of education and more loneliness. General health also correlated with age, with increasing age being associated with a decrease in self-reported health. Neither mental health nor loneliness was related to inhabitants’ age.

**Table 2:** Correlations (Kendall t) between demographic and health variables

	1	2	3	4	5		6
1. Loneliness	1						
*N*	995						
2. Age	0.04	1					
*N*	956	993					
3. Education	− 0.13*	− 0.33*	1				
*N*	995	993	1036				
4. Income	0.26*	0.09*	− 0.29*	1			
*N*	984	977	1019	1019			
5. General health	0.24*	0.13*	− 0.13*	0.24*	1		
*N*	992	988	1030	1016	1030		
6. Mental health	0.38*	− 0.00	− 0.10*	0.25*	0.49*	1	
*N*	994	976	1018	1004	1014	1018	
7. Health literacy	− 0.26*	− 0.16*	0.19*	− 0.19*	− 0.21*	− 0.22*	
*N*	910	893	923	916	920	919	

* *p* < .001

**Fig. 3:**
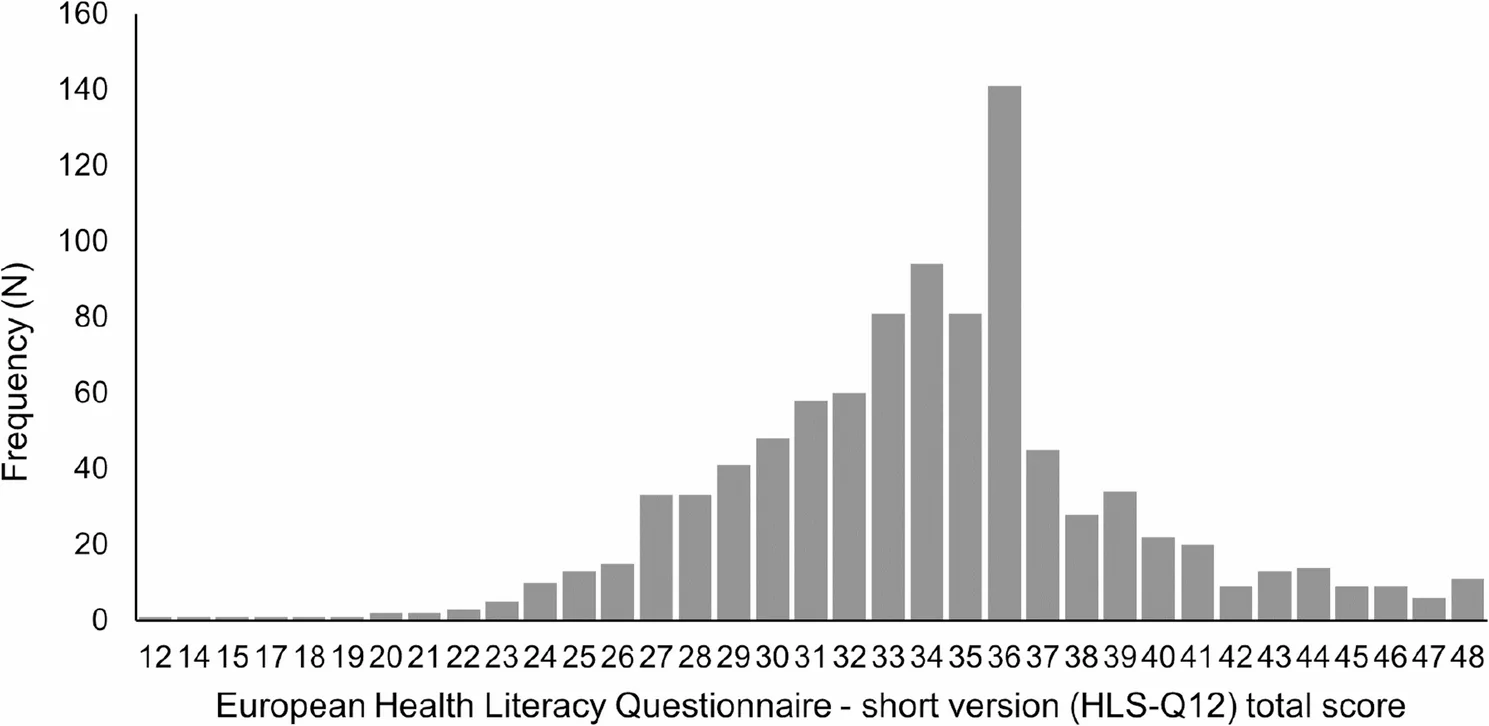
Health literacy scores (*N* = 945)

The sample obtained a mean HLS-Q12 health literacy score of 34.04 (*SD* = 5.13; Fig. 3). Health literacy showed a positive correlation with education and correlated negatively with loneliness, age, income, general health, and mental health (Table 2). The correlations indicated that lower health literacy is associated with lower household income, older age, poorer general health, poorer mental health, and higher levels of loneliness. On average, women scored higher in terms of health literacy compared to men (*F*(1,936) = 8.60; *p* = .003).

#### Demographic factors predicting health literacy

Independent variables were included in the hierarchical regression for the prediction of health literacy in 5 steps (Table 3). Assumptions of multicollinearity, normality of residuals, and homoscedasticity were not violated. Model 4 provided the best fit, explaining 16.3% of the total variance in health literacy. Age, education, general health, mental health, and loneliness are unique predictors for health literacy, whereas no additional contribution was found for gender or income. The direction of effects showed that health literacy was lower in individuals with higher age, lower education, lower general and mental health, and higher scores for loneliness.

**Table 3:** Hierarchical regression analysis for the dependent variable health literacy

	*R*²	*R*²_adjusted_	DR²	Standardised β-coefficient
Model 1	0.07	0.07	0.07**	
Age				-0.16**
Gender				-0.04
Education				0.18**
Model 2	0.10	0.10	0.03**	
Age				-0.14**
Gender				-0.04
Education				0.17**
General health				0.17**
Model 3	0.14	0.13	0.03**	
Age				-0.15**
Gender				-0.05
Education				0.16**
General health				0.08*
Mental health				0.20**
Model 4	0.16	0.16	0.03**	
Age				-0.15**
Gender				-0.05
Education				0.15**
General health				0.07*
Mental health				0.15**
Loneliness				-0.17**
Model 5	0.17	0.16	0.003	
Age				-0.15**
Gender				-0.05
Education				0.14**
General health				0.06
Mental health				0.14**
Loneliness				-0.16**
Income				0.06

*R*² is the proportion of variance explained by the model. *R*²_adjusted_ is an estimation of the variance explained on population level. DR^2^ verifies whether a model is a significant better fit compared to the previous model. Standardised β shows the unique contribution of each variable. *p < .05; ** *p* < .001

#### Requirements for primary care and prevention

Individuals considered health to be very important (*M* = 9.40 out of 10, *SD* = 1.04, *N* = 1060) and most had a regular family doctor or GP (96.9%; *N* = 1021). However, this GP was located in the municipality for only 33.7% (*N* = 344) and the GP was even situated beyond neighbouring communities for 9.5% of community members (*N* = 97). Factors that played a role in GP selection were (ranked from most to least important): (1) being able to access the GP quickly (mean rank 1.54), (2) physical distance (mean rank 2.3), (3) recommendations from friends or family (mean rank 3.47), (4) GP as part of a primary care centre (mean rank 3.72), (5) other (mean rank 3.97). Common self-reported other factors consist of having a relationship of trust with the GP (*N* = 137), GP competency (*N* = 81), GP empathy (*N* = 45), communication skills (*N* = 29), and providing sufficient time for patients (*N* = 26).

Participants generally stated that it is easy (73.0%; *N* = 734) or very easy (21.9%; *N* = 220) to comprehend their GP. A total of 4.6% (*N* = 46) reported that their GP is hard to understand and 0.5% (*N* = 5) indicate it is very hard to understand the GP. Understanding one’s own GP was closely related to one’s health literacy (*t* = 0.39; *p* < .001; *N* = 942). There was also an association between age range and the extent to which respondents indicated they understood their GP (*t* = -0.07; *p* = .02; *N* = 965), which is in line with lower health literacy skills with increasing age. The statement “I have at least one caregiver by whom I feel understood and supported” (0 not at all agree − 10 completely agree), had a mean score of 7.77 (*SD* = 2.40; *N* = 1009). The top 3 preferred additional disciplines in the primary care centre were dentist, nurse, and psychologist (detailed findings can be found in Supplementary file 1).

In terms of prevention, the vast majority of the population considered a healthy lifestyle important (*M* = 8.61 out of a possible 10; *SD* = 1.43; *N* = 1031). Participants ranked several lifestyle factors according to the influence they could have on health outcomes. Table 4 shows the results of the Friedman ANOVA, *X²* = 2985,71; *p* < 0,001; *N =* 920. Healthy food was perceived as the most important factor regarding health prevention, followed by physical exercise. Exploratory analyses showed only minor differences in ranking across age groups. People over 80 years attributed a bit more influence to sleep compared to mental health and individuals under 60 years ranked smoking before alcohol consumption. Other factors proposed by participants were social contact/network (*N* = 20), the environment (quiet and air pollution; *N* = 11), illnesses (including drug addiction, *N* = 6), medical follow-up and counselling (*N* = 6), activities/meaningful use of time (*N* = 4), finances (*N* = 4), sexuality (*N* = 3), work/work-life balance (*N* = 3), heredity (*N* = 3) and a residual category (*N* = 6).

**Table 4:** Participants’ ranking of the extent to which certain lifestyle factors influence health

Health factors	Mean rank
Healthy food	2.05
Physical exercise	2.62
Mental health	3.43
Sleep	3.55
Alcohol consumption	4.71
Smoking	4.87

Lower mean rank represents higher perceived influence on health

On a scale of 0 (not at all) to 10 (definitely yes), participants reported a mean score of 6.32 (*SD* = 3.05; *N* = 983) on willingness to use a health tool, such as a website or app, to measure their health and take preventive actions in terms of health. Exploratory analyses show that willingness to use such digital tools decreases with age (*t* = -0.15; *p* < .001, *N* = 949). In the oldest age group, the willingness to use digital tools in the health context is significantly lower (*M* = 3.17; *SD* = 3.59; *N* = 82).

Most residents believed that the local government has a role to play in health prevention. This was reflected in a score of 7.10 (*SD* = 2.36, *N* = 1018) on an 11-point rating scale (0 = *not at all*; 10 = *definitely*). An open question regarding this topic revealed that motivating, stimulating, and activating residents is the most important task (*N* = 93), followed by informing people on prevention through thematic sessions, providing health articles in the local newspaper, etc. (*N* = 78). In third place, they mentioned the need to involve a sufficient number of affordable GPs, dentists and paramedics in the primary care centre in order to make health (promotion) accessible to all inhabitants (*N* = 73). Some other categories that were mentioned frequently include inclusive policies (*N* = 20), financial support (*N* = 19) and provision of a confidential advisor (*N* = 15).

### Interim discussion

The survey study reached a substantial sample and a comparison with government statistics showed that the sample was a relatively good cross section of inhabitants of the municipality, including elderly and residents in a situation of vulnerability. Findings indicated that the citizens were in reasonably good health. Lower general and mental health were related to lower income, lower levels of education and more loneliness, which is consistent with previous research [27–29].

The sample showed varying levels of health literacy with an average score of 34. Previous work has suggested that people with a score upwards of 26 generally have access to, understand and can apply information important for staying healthy [30]. When reaching 33 or higher, additional skills are added, namely finding, understanding, assessing, and applying health information and advice relevant to improving physical and mental health. Individuals with a score of 39 and above have all the previous skills and are able to make informed health choices by critically evaluating health-related statements and making informed comparisons between different treatment options. Higher age, lower education, lower general and mental health, and higher levels of loneliness predicted lower health literacy. This is in line with previous work which shows that education, language proficiency, income, age, illness and emotional problems are factors that impact health literacy [16, 31–33]. Reduced health literacy in older individuals has also been documented before [34, 35] and could be linked to the observed lower willingness in elderly to use digital tools for health promotion (although personal preference may also play a role).

Findings support a need for accessible multidisciplinary primary care centres including GPs, dentists, nurses, and psychologists. Additionally, citizens endorsed the local government to play a role in health promotion related to e.g., healthy food, exercise, and mental health.

While findings regarding health literacy, (mental) health and related characteristics are in line with existing evidence, other findings and preferences will likely be driven by the local context and cannot be generalized beyond this municipality. There are also some other limitations that require mentioning. Since no data was collected on ethnicity, we cannot be sure whether we were able to cover the ethnic diversity of the population. In the paper survey (mostly completed by elderly participants), the health literacy questionnaire was regularly skipped. This could indicate that the survey might be too difficult for some, and the results could be an overestimation of health literacy skills in this population. A final limitation concerns the cross-sectional nature of the study, which precludes conclusions on causal directionality.

## Study 2: collaborative primary care design

Since study 1 shows that citizens endorse the local government initiatives and healthcare organisation study 2 provides an overview of different activities and actions to support the quality of integrated primary care in the community, including a primary care centre and its activities. These activities also took place in the municipality of Vorselaar (Belgium) where the local government wanted to co-design a new health policy with a strong focus on health prevention and multi-actor collaboration. A multidisciplinary primary care centre (Gezondheidshuis PIOEN) was opened in October 2022. The living lab collaborated with the municipality to facilitate several activities (Fig. 1). A first set of co-creation sessions focused on primary care needs. This was followed by a survey on health, lifestyle and prevention. Since low involvement in the existing preventive offer was identified in the survey, additional co-creation sessions were performed with vulnerable residents to explore possible motivators and barriers to participate in prevention initiatives organised by the municipality. The activities were approved by the Social and Societal Ethics Committee SMEC of KU Leuven (G-2022 06 2111) and all participants provided informed consent.

### Method

#### Co-creation on primary care needs

##### Recruitment

For the co-creation sessions on primary healthcare needs, recruitment aimed to include citizens, care professionals, and vulnerable residents. We aimed to include about eight to ten individuals per group, with a minimum of four individuals for the samples that are more difficult to reach (in line with Carlsen & Glenton [36]). Citizens were recruited by the panel managers of the living lab through targeted emails to respondents of the survey from study 1 who had indicated to be willing to participate in follow-up research. Batches of approximately 10 emails were sent every two days until a satisfactory number of participants for the sessions was reached. We aimed to include a balanced ratio of males/females and participants of different age groups. All care professionals active in the municipality were invited for participation by the local government through email. Finally, more vulnerable residents (e.g., with low education levels and expected limited health literacy) were recruited with the help of an intermediary local organisation.

##### Activities & materials

**Fig. 4:**
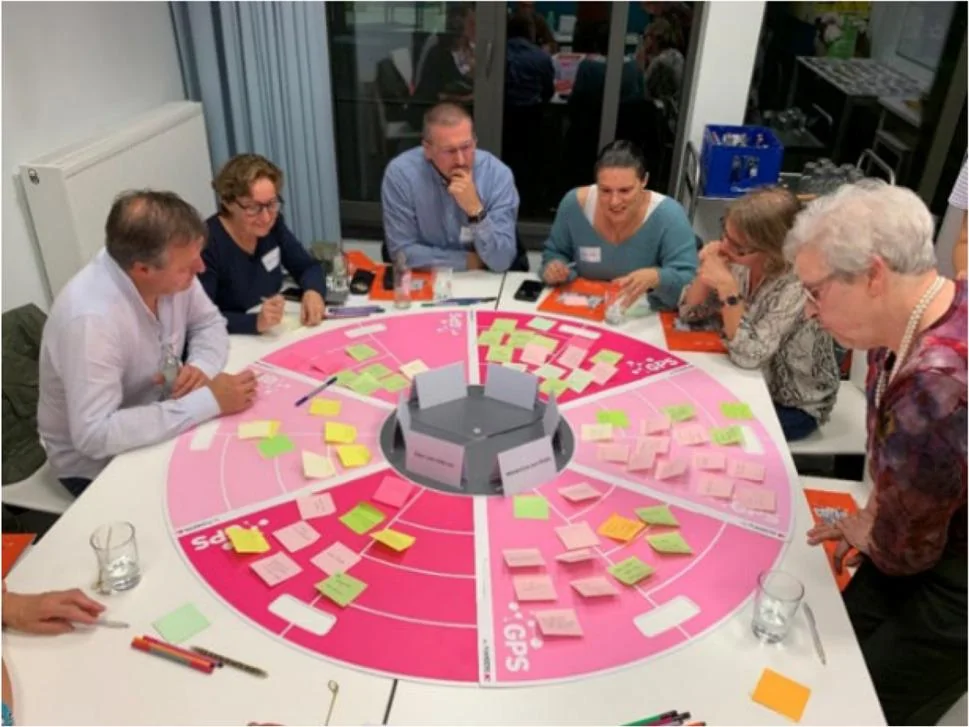
The GPS brainstorm kit as co-creation template

The goal of these co-creation sessions was to generate ideas for the future local health strategy. Every co-creation session started with an ice-breaker game (to make participants more conducive to creativity and sharing, and relax the atmosphere). Subsequently, both the primary care centre and the living lab were introduced after which the GPS brainstorm kit ([37], Fig. 4) for idea generation was used in all three sessions. GPS brainstorm is a structured brainstorming method in which five relevant subthemes related to the vision and strategy of the upcoming multidisciplinary healthcare centre were presented. For the citizens, these themes were preventive health, to inform and learn, being open for everyone, being an inviting meeting place, and the design of the new building which would house the centre. For the care professionals the focus was a little different and included the themes preventive health, primary healthcare, enhancing health literacy, multidisciplinary, and partner network. Additionally, a sixth residual subtheme or free field, in our sessions called ‘the sky is the limit’, encouraged participants to think out-of-the box and to come up with innovative ideas without restrictions in available resources. The generated ideas were written on sticky notes individually and subsequently prioritised in group.

##### Analysis

The analysis of the co-creation sessions was based on the written and orally generated ideas collected during the GPS brainstorm activities. For each session, all ideas were first organised according to the predefined themes. Within each theme, the ideas were then summarised into broader categories, focusing on recurring suggestions, shared concerns, and concrete action points for the future local health strategy. The analysis thus combined a deductive structure, based on the predefined session themes, with an inductive synthesis of the specific ideas and priorities raised by participants. For the sessions with citizens and care professionals, the analysis drew on the individual ideas written on sticky notes and discussed and prioritised in the group. For the session with residents with limited health literacy, the same thematic structure was used, but the procedure was adapted because two participants had severe visual impairments. In this session, the themes were discussed orally in the group, and the analysis was based on the ideas generated during this collective discussion. The results were analysed and reported separately for the three participant groups and per theme, after which they were used to identify concrete implications for the development of the multidisciplinary primary care centre. A synthesis of the general co-creation results is presented in the current paper.

#### Survey on health, lifestyle and prevention

##### Recruitment

Recruitment was performed in three ways: distribution of an information flyer with QR code and web link to the survey (e.g., on the local annual fair, at the newly established primary care centre PIOEN), communication by and through the local administration (e.g., through the municipal bulletin, social media) and a personal invitation via e-mail from the living lab (i.e., participants of study 1 who wanted to stay involved in local research activities). Participants could complete the survey between June 17, 2023 and August 31, 2023. There was a possibility to fill in a paper questionnaire (available at several public locations such as the primary care centre and local service centres) or an online questionnaire. A total of 286 questionnaires were collected online (*N* = 284) or on paper (*N* = 2). This corresponds to a response rate of 4.5% of the adult population in the municipality. However, five participants did not answer substantive questions after completing their profile information and were excluded.

##### Activities & materials

The survey focused on participants’ health, lifestyle, and the topic of prevention. The first aim was to gain a deeper understanding of the physical, mental and social health of the residents and any implications on their daily activities. Physical health refers to bodily health and physical fitness. Mental health refers to psychological well-being. Social health refers to satisfactory social contacts and participation in activities in the community. Second, different aspects of a healthy lifestyle such as nutrition, exercise, sleep, alcohol consumption and tobacco use were addressed. Third, we addressed the topic of health prevention. The local community regularly organizes health prevention activities and promotes such activities through articles in the municipal bulletin, on social media and on the website of the local primary care centre. The survey assessed which articles participants read and which activities they already participated in between January and May 2023. The survey also inquired about interesting health topics, possible motivators for participation, appropriate communication channels, use of digital tools, and suggestions for health prevention. The survey contained items from validated national or internal instruments, such as the Dutch-language SF-RAND-36 (Short Form Health Survey [38]) for quality of life, AUDIT (Alcohol Use Disorder Identification Test [39]) questions on alcohol use, and other nationally developed instruments [40, 41]. Some questions were directed according to gender and age (e.g. concerning cervical cancer). Completing the questionnaire took about 10–15 min. A translated version of the full questionnaire can be found in Supplementary file 2.

##### Analysis

Responses were analysed with SPSS 29 or through thematic analysis for open-ended questions. Sample sizes can vary between analyses due to incomplete responses. Therefore, sample sizes will be reported per analysis.

#### Co-creation on prevention activities for residents in a situation of vulnerability

##### Recruitment

For the co-creation sessions on motivators and barriers to participation in preventive health activities, the panel managers specifically aimed to recruit citizens of Vorselaar with lower health literacy and from more vulnerable groups. Recruitment took place through (a) posters and flyers placed at and distributed by organisations targeting more vulnerable individuals (e.g., the local service centre (“Sprankel!”), a local welfare and poverty organisation (“‘t Kiertje”), social grocery distribution centre); (b) presenting the research at the community restaurant and personally motivating individuals to participate; (c) inviting more vulnerable participants from the survey study.

##### Activities & materials

This set of co-creation activities started with a brief explanation of the purpose and course of the group discussion. The preventive health activities of the municipality were presented and participants ranked the activities on a scale from 1 (I would absolutely not participate in this) to 10 (I would really like to participate in this) to facilitate discussions. Next, participants received templates to note and discuss (1) motivators for participation, (2) barriers for participation, and (3) how to motivate more people for participation. Finally, the participants looked for ways to overcome these barriers and motivate more people to participate in these preventive health activities. All participants signed an informed consent before the session started. After the session, the participants filled in an evaluation. The group discussion lasted a maximum of two hours. Data was collected through post-its, templates and session notes by an observer.

##### Analysis

The analysis of the co-creation sessions was based on multiple sources of session material, including facilitators’ notes, the transcript of the group discussions, completed templates and post-it notes. During the sessions, participants first rated a set of existing preventive health activities on a scale from 1 (“I would definitely not participate”) to 10 (“I would very much like to participate”). These ratings were used as a starting point for a qualitative discussion of reasons for participation or non-participation. The analysis followed the structure of the session guide and focused on three main analytical categories: motivators for participation, barriers to participation, and strategies to encourage broader participation in preventive health activities. Within each category, the collected ideas and reflections were marked and grouped. Similar responses were then clustered into broader themes.

### Results

#### Co-creation on primary care needs

##### Citizens

Ten citizens participated in the co-creation session. Participants indicated that the local government should target health promotion to inhabitants of all life stages, starting at school. The primary care centre (PIOEN) should be easily accessible and affordable for all citizens. Different online and offline contact options should be available flexibly (daytime, evening, weekend). The primary care centre is recommended to not only accommodate doctors and paramedics but also facilitate the hosting of workshops or thematic sessions regarding health-related topics. Examples consist of yoga or work-out sessions, workshops to enhance individuals’ computer/digital skills, social media training, and first aid trainings. Participants also had several suggestions concerning the design of the new building. Citizens recommended to include plenty of natural light, art of local artists, a children’s corner, a stable internet connection, a large parking lot nearby, and some benches for informal talks.

##### Care professionals

Fifteen local care professionals from nine different disciplines participated in the co-creation session (out of 51 municipality care professionals; response rate 29%). They acknowledged that cooperation across disciplines was limited at the time. Online information on local care professionals was often outdated and they, therefore, felt the need to get acquainted with each other and each other’s expertise. Better interrelations would also facilitate referral between the disciplines. One suggestion was to build an online platform with updated information of all care professionals of the municipality. Care professionals wanted to prioritise inclusion of residents in a situation of vulnerability but found it a challenging subject to tackle on their own. Therefore, they suggested to get together quarterly to exchange ideas on different health themes, including how to strengthen residents’ health literacy. However, one concern that emerged was the funding of health promotion activities. Dissemination of events or thematic sessions could use the proposed platform or website of the primary care centre. Participants also stressed the importance of attracting enough dentists for the primary care centre, as the municipality had lacked a dentist for several years already. Finally, targeting schools for health prevention was considered an important step to start early with the introduction of healthy life habits. This entails the additional advantage that school children can act as a gateway for providing information to parents who might be more difficult to reach, such as parents with a migration background or lower socio-economic status.

##### Residents in a situation of vulnerability

Five residents in a situation of vulnerability could be recruited for a co-creation session. One of the coordinators of the intermediary social organisation also participated in the session. Participants indicated that the best way to improve accessibility for residents in a situation of vulnerability is through personal contact. However, these people often tend to live in isolation. Since doctors are not allowed to advertise their services (in line with the Belgian law), the participants suggested that volunteers affiliated with the primary care centre could go door-to-door to inform about their services. These volunteers could work with informal local leaders who are aware of specific difficulties in the neighbourhood. In terms of the design of materials, leaflets for health prevention activities should be playful rather than pedantic, with concrete tips and tricks. Preventive initiatives could be conceptualised as group challenges that are rewarded. Furthermore, participants also indicated that the primary care centre should be easily accessible, e.g., for wheelchairs or rollators, and touch screens at the entrance should preferably be avoided as they are a burden to the visually impaired. The centre should have extensive opening hours but additionally, offer GP home visits since they are essential for those who are less mobile. One should be able to make an appointment online or by phone. A warm welcome was deemed important as they indicated that the primary care centre should have a cosy reception with friendly staff that help without prejudice. The idea of a coffee corner as a meeting place was also suggested. Finally, the participants would like the primary care centre and the local government to organise workshops for citizens to improve both digital and health literacy skills.

#### Survey on health, lifestyle and prevention

**Fig. 5:**
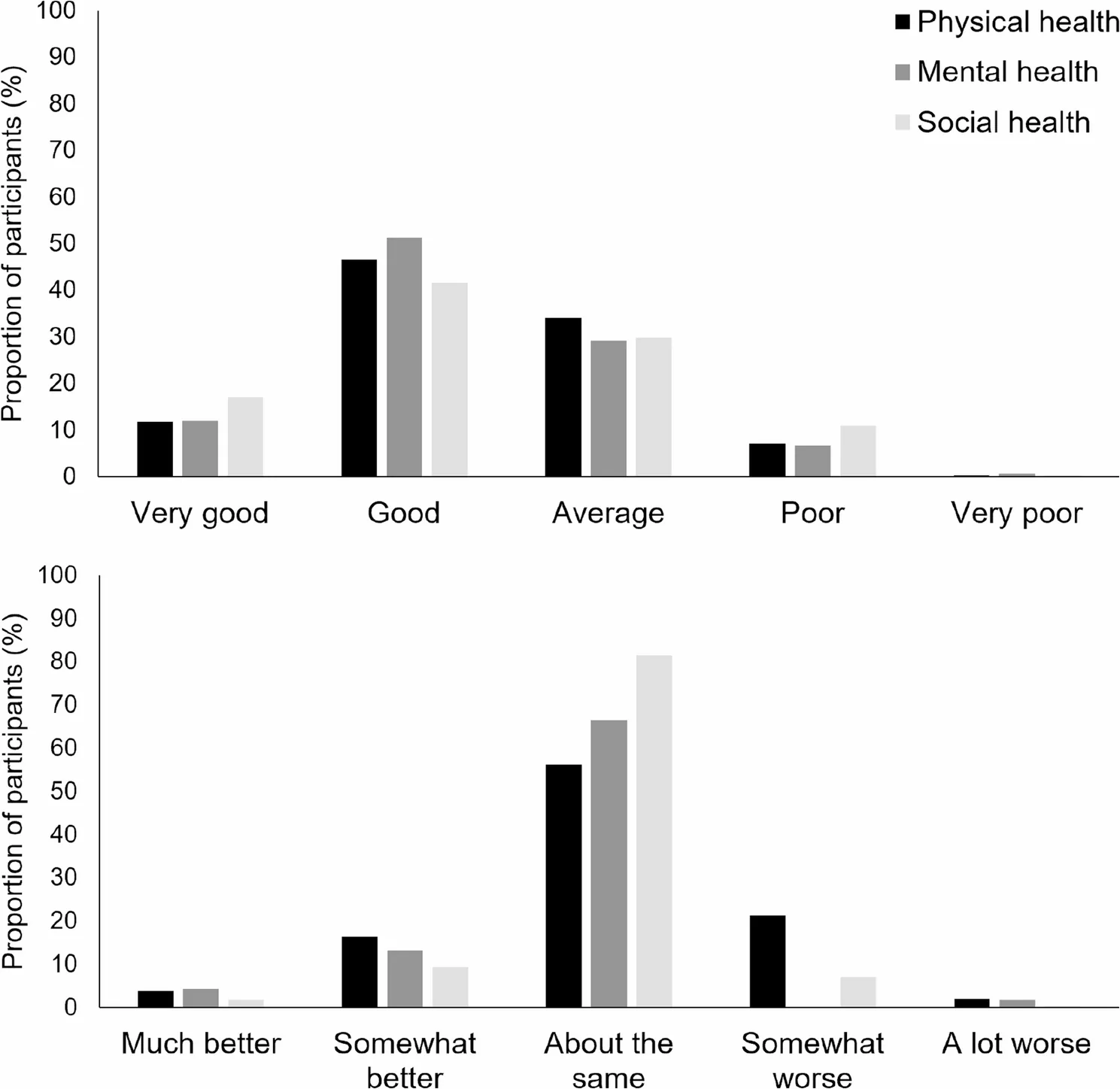
Physical, mental and social health (top) and the changes in health in the past year (bottom) (*N* = 281)

An overview of demographic information of the 281 participants can be found in Table S2.1 of supplementary file 2. About 8.5% of respondents (*N* = 24) report some difficulties to a lot of difficulties in making ends meet and can be considered residents in a situation of vulnerability. Almost 1 in 3 respondents (*N* = 89) mentioned suffering from a chronic condition with an average impact on daily life of 5.7 (*SD* = 2.37) on a scale of 0 (not at all) to 10 (very much). More than 3 in 4 of all respondents (76.4%; *N* = 215) reported being able to perform daily activities such as walking, climbing stairs, carrying groceries or moving a chair without difficulty. Overall, physical, mental and social health was rated average or above (Fig. 5). About 7.5% of residents are in (very) poor physical (*N* = 21) or mental (*N* = 21) health and around 11.4% are in (very) poor social health (*N* = 32). Social health seems the most stable, with 81.49% of people (*N* = 229) experiencing no changes over the past year. On the psychological and physical level, respectively 66.6% (*N* = 187) and 56.2% (*N* = 158) participants indicate that they still feel about the same as they did a year ago. Changes in physical, mental and social health are fairly evenly distributed between positive and negative changes.

**Fig. 6:**
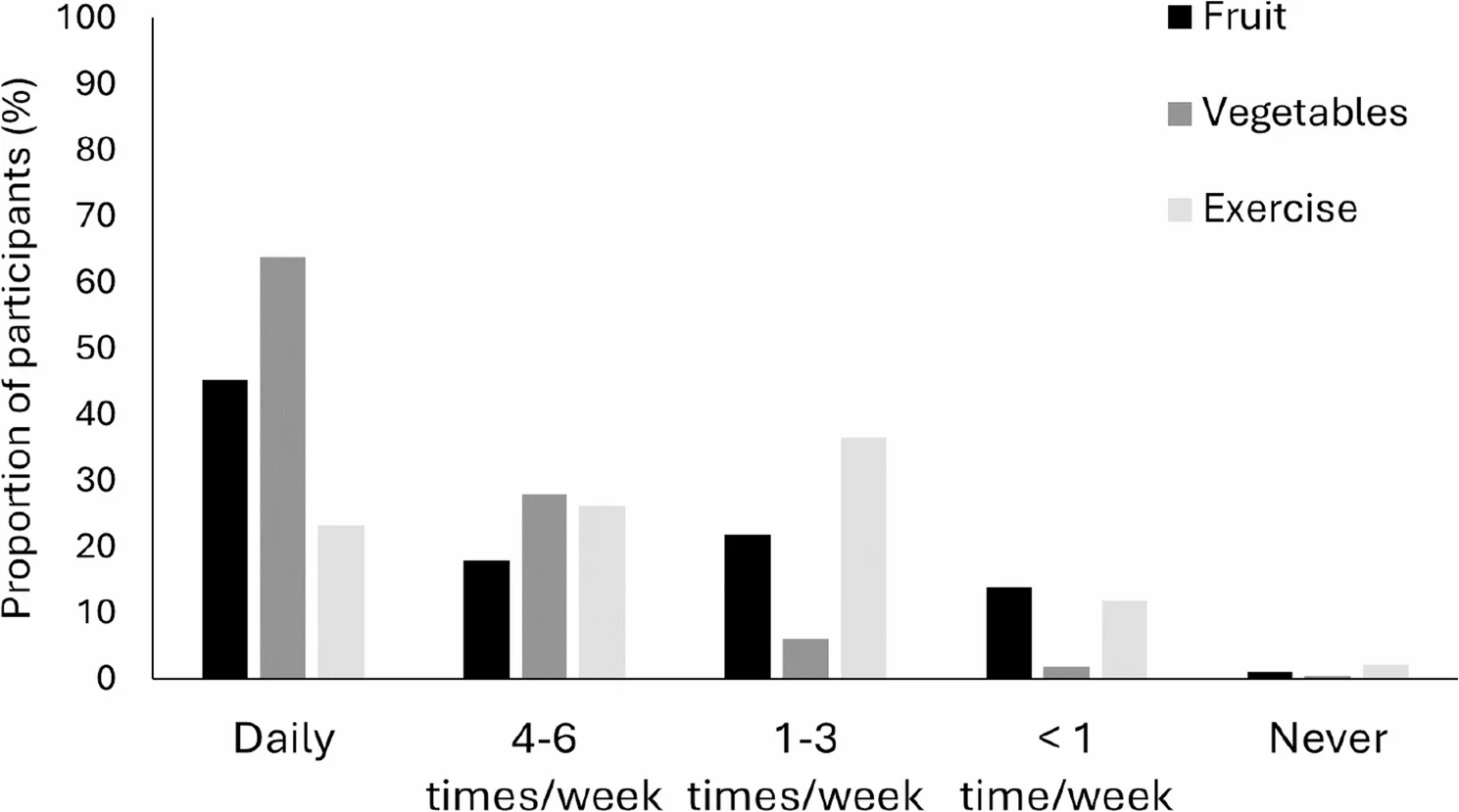
Frequency of consuming fruit and vegetables and exercising (*N* = 279)

Figure 6 shows consumption of healthy foods, especially fruits and vegetables, and physical exercise. Over 1 in 3 participants (*N* = 103) eat fruit less than four times per week. Consumption of vegetables is substantially higher. About half of the respondents exercise at least 4 times a week for 30 min in addition to their daily activities (*N* = 138). A minority of 14.0% (*N* = 39) moves less than once a week, with 2.2% (*N* = 6) never exercising.

Concerning sleep, about 1 in 5 reports often (19.0%; *N* = 53 ) or (almost) always (2.9% *N* = 8 ) experiencing difficulty falling asleep. More people appear to have trouble staying asleep than falling asleep. For example, 1 in 3 participants reported often having trouble sleeping through the night (*N* = 93), and for another 12.2% of residents this is (almost) always a problem (*N* = 34). More than half of participants (52.3% *N* = 146) sometimes feel tired during the day. For 1 in 5 this is even often (17.2%; *N* = 48) or (almost) always the case (2.9%; *N* = 8).

Over 1 in 3 respondents do not consume alcoholic beverages. Of those who do consume alcohol (*N* = 175), about 21.7% (*N* = 38) drinks at least four times per week, 34.9% (*N* = 60) consumes alcohol two to three times a week, 35.4% (*N* = 62) consumes alcohol two to four times a month, and 8.0% (*N* = 14) drinks monthly or less. About a third of individuals who consume alcohol, drinks at least three standard glasses at a time (*N* = 63). 10% of alcohol consumers drink more than five standard glasses (*N* = 17), of which five participants drink at least seven standard glasses at a time. At the time of sampling, 93.2% of respondents said they did not smoke cigarettes, cigarillos, cigars, or e-cigarettes (*N* = 261). Just under half of these individuals did smoke in the past (*N* = 120). More than half of the smokers (*n* = 9/17) are thinking of quitting smoking.

When it comes to current prevention initiatives of the community, over half of participants (*N* = 148) did not read communications on health prevention from the municipality or participated in activities organized. The majority of respondents within the recommended age range had been screened for breast and/or colon cancer in the past two years (78.7%, *N* = 75; 77.0%, *N* = 152, respectively). Similarly, about 4 in 5 women between the ages of 25 and 64 (79.7%; *N* = 123) indicated that they had been screened for cervical cancer in the past three years. The local primary care centre also offers a prevention website, which was visited by about 29.9% of participants (*N* = 274). Participants could indicate thematic themes of interest and prime themes were diet, physical activity, sleep, mental health, dealing with stress (Table S2.2 in supplementary file 2). However, most participants were not interested in participating in such or other health or prevention initiatives from the local government, local associations, or the primary care centre in the future.

The main motivators for participating in health prevention initiatives were time of day (*N* = 52 with preferences for both evening (*N* = 28) or daytime (*N* = 17) activities), cost (*N* = 44) and nearby location (*N* = 39). Other regularly mentioned motivators were the type and difficulty level of the activity (*N* = 18), whether it was in group (*N* = 11), whether it was child-friendly (*N* = 10), and practical feasibility (*N* = 7). Information about upcoming preventive initiatives should be communicated through the monthly municipal magazine (available online and on paper) (*N* = 214), flyers in the letterbox (*N* = 115), or social media (*N* = 92). More detailed information about communications about, preferences for, and attendance to health or prevention initiatives can be consulted in supplementary file 2.

#### Co-creation on prevention activities for residents in a situation of vulnerability

Four sessions included a total of 21 citizens (5 male, 16 female). Participants were between 34 and 89 years old (with the vast majority over 60 years old). Participants varied in digital skills from no experience with digital tools to more substantial experience with computers, mobile phones and social media. Table 5 provides an overview of what participants indicated as important motivators for participation, and includes, social, personal and practical motivational factors.

**Table 5:** Motivators for participation in preventive health activities

Motivator	Explanatory notes
Social interaction	Participants enjoy getting to know new people from the neighbourhood, exchanging ideas, and being together.
Eagerness to learn and curiosity	People like to learn, stay informed, gain insight and get inspired about health-related topics.
Facilities and mobility	In terms of the physical location, good facilities and good mobility are important. Participants prefer accessible and drop-in (non-binding) activities.
Price	As far as the price is concerned, participants find it important that activities are free or affordable.
Accompaniment	A good activity facilitator who gives a clear explanation and uses a personal approach can encourage people to participate.
Intrinsic motivation and personal beliefs	Not only the nature of the activity, but also intrinsic motivation and personal beliefs determine whether or not someone will participate in preventive health activities. People who put their health first, who take care of themselves, who enjoy exercise and who value the usefulness of prevention, will be more likely to participate in activities.
Pleasure	A relaxed atmosphere, good food, music, dancing, and fun events and activities appeal to the participants. There should also be sufficient variety in activities.

The barriers for participation are reported in Table 6. Several themes strongly align with the motivators, for example regarding facilities and mobility, but new topics focus on for example communication and digital skills.

**Table 6:** Barriers to participation in preventive health activities

Barriers	Explanatory notes
Facilities and mobility	Mobility and facility problems can be barriers to participating in activities (e.g., heavy traffic, uneven footpaths).
Price	An elevated cost of activities or healthy food can prevent people from living a healthy life.
Intrinsic motivation and personal beliefs	It was acknowledged that it can be difficult to change your lifestyle, break certain patterns and adjust your habits, and that intrinsic motivation is relevant in this respect.
Communication and information	Limited communication and information about the activity or not knowing exactly what the activity entails or what the added value is, can also hold people back.
Physical barriers	Physical barriers can prevent people from participating (e.g., not being identified as part of the target group, not being mobile enough to participate, or having food restrictions preventing you from taking part in a healthy cooking activity). Activities can also be difficult to combine with people’s work rhythm.
Social barriers	Social barriers such as there being too many people, having to go somewhere alone, too little privacy during sports, not knowing many people from the community yet, can also make it harder to participate.
Digital barriers	Activities that call on people’s digital skills received mixed reviews. Some people have difficulty with the multitude of applications and the speed of digitization.

Building on these motivators and barriers, several strategies for improving participation in preventive health activities were suggested. These are presented, according the theme, in the following paragraphs.

##### Communication and information

Motivating individuals to participate firstly consists of properly informing more people about the activities. This can include word-of-mouth advertising (a human point of contact, personally addressing individuals), more promotion about the activities (posters, social media, electronic information sign a busy community square or location) and potentially working with an ambassador per neighborhood. The monthly community magazine can be used to promote activities but also to disseminate health and prevention information directly. An accessible hotline can encourage people to report both physical and mental barriers to participation so that activities can be adjusted.

##### Overcoming social barriers

Social barriers can possibly be overcome by ‘bring a friend’ initiatives, by focusing on community work, and by using experts by experience as intermediaries. Preventive health activities should ideally appeal to all ages and could be organised at different times and throughout the year so that everyone has the opportunity to participate. Additionally, it was indicated that it is relevant to pay additional attention to single, working women.

##### Focusing on a wide range of topics and actions

Participants provided suggestions to expand the offer further. The municipality can encourage residents to propose initiatives themselves and can support such new initiatives. The output of the survey could be of help for the identification of themes (see also supplementary file 2).

##### Overcoming psychological barriers

To give people a psychological boost, participants suggest giving a reward for participation. Attention should also be paid to ways to visualize progress or perseverance. Activities could be suggested to specific individuals if there is a need (e.g., by the GP). However, people with disabilities want to be treated as ordinary people and don’t want to be patronized but empowered. A positive approach is important the activities should focus on health and not on illness. Sufficient attention should be paid to encouraging people to open up to new things and to the fact that a change in behavior can be difficult to achieve.

##### Overcoming physical barriers

To overcome physical barriers, people are thinking of adapted activities for people with a physical disability. For people with allergies and intolerances, information sessions and an adapted cooking course could be provided.

##### Mobility

To ensure that potential participants can easily travel to the activity, one should focus on mobility. Furthermore, the proximity of sanitary facilities is important (for example during a walk). Activities can also be linked to popular events such as a fair (in addition to a stand-alone offer). Not needing to register in advance can lower participation thresholds.

### Interim discussion

Study 2 provides an overview of several participatory activities which were implemented to guide the design of local health policy with a strong focus on health prevention and multi-actor collaboration. By combining multiple perspectives and research activities, the current study was able to gain rich insights allowing for the design of better primary healthcare and prevention in the community. The study succeeded in including almost one third of local care professionals in co-creation. Co-creation sessions revealed that both citizens and care professionals were willing to invest in the municipality’s health landscape and led to suggestions for concrete actions for concerning the primary care centre as well as preventive activities organised by the local government.

The survey suggests that, in general, the health of the municipality’s residents is satisfactory. Of the three health aspects (physical, psychological and social), social health received the lowest ratings with over 1 in 10 residents reporting to be in (very) poor social health. Social health also appears to be the most stable with over 8 in 10 persons experiencing no changes over the past year. In terms of lifestyle, sleep quality appeared satisfactory but tiredness during the day was common. In terms of alcohol and tobacco use, the sample is more healthy than the Belgian average. For example, more than one-third of our respondents reported no alcohol use, compared to only 23% on average in Belgium [42], and only 16 individuals were smokers. Despite its obvious negative effects on our bodies and health, alcohol consumption is socially embedded in our society [42].

Such lifestyle choices and behaviours can promote health and be relevant targets for prevention. Research has continuously underscored the relevance of nutrition and exercise [43, 44], sleep [45–47], and the use of alcohol and other addictive substances. alcohol use affects almost all organs and is associated with 60 different disorders [48, 49]. People who consume alcohol or other stimulants do not often report directly to a general practitioner or other health care professional with this complaint. Often problematic use becomes evident through indirect signs, such as fatigue, sleep problems, and stress-related complaints [50]. When survey participants were asked about their lifestyle interests, in line with what was expected based on the literature, diet, exercise and sleep were included in the top 5. Additionally, mental health and stress were deemed important. Previous research has also underscored the high prevalence of stress and mental health burden as well as an increasing interest in this topic [51].

Campaigns for preventive screenings, organised by the government, are showing promising results. Over three-quarters of respondents in the target groups report undergoing screenings for breast, colon, and cervical cancer. However, prevention initiatives and informative health-related articles disseminated between January and May 2022 through various channels, including the municipal information sheet, website, and social media, did not reached the desired audience. About a third of the respondents had already found their way to the preventive website of the local primary care centre, but less than half of the respondents were open to participating in activities organized by the municipality or other local actors. Future studies should assess whether this is caused by a lack of actual or perceived need or a lack of interest in the offer.

Survey results were extended with additional co-creation sessions with citizens in a situation of vulnerability. Participants in these sessions were positive towards the new multidisciplinary primary care centre. The participants had several suggestions for improvements, such widening the scope of activities, providing additional support for lifestyle changes and helping to build intrinsic motivation, considering mobility problems, and paying attention to the reach and potential barriers to participation. They also consider it important to make room for trying out new activities and to evaluating and adjusting new and existing activities if necessary, a dynamic process which could be assisted by working with neighborhood ambassadors. ‘Bring a friend’ initiatives and the use of enthusiastic participants and experts by experience can motivate citizens to participate in preventive health activities. The general practitioner is also viewed as a central person with a holistic view of citizens. Therefore, they are in a good position to link the citizen to preventive health activities that are personally tailored to the specific needs of the citizen.

## General discussion and conclusions

Primary care benefits from being organized according to community and stakeholder requirements. Such a redesign requires insight into the health literacy, preferences and needs of the community and it benefits from a participatory approach. The current work describes a partnership between a living lab and a local government to organize an inclusive and large-scale stakeholder consultation for healthcare reorganization. Generalizable insights into community health and health literacy were combined with a participatory approach to help redesign primary care and prevention. Important outcomes will be discussed linked to four domains: community health, impact on community care redesign, the added value of the ‘living lab’ approach, and implications for policy and practice.

### Community health

Participating citizens (*n* = 1078) were in reasonably good health and generally had satisfactory health literacy. Several demographic factors appeared to be associated with risks in terms of health and health literacy. Firstly, there was a negative association between health literacy and health itself. A separate relevant factor was education level, which was associated with health literacy but also general and mental health. Loneliness also proved to be an important social factor contributing to risk in general health, mental health, and health literacy. Income appeared to be mainly important for general and mental health, while age was important for health literacy. As mentioned in the interim discussion, some other work has also underlined the importance of these respective risk factors for health [27–29] and health literacy [16, 31–35]. Survey findings supported a need for accessible multidisciplinary primary care centers and citizens endorsed the local government to play a role to play in health promotion and community care.

### Community care redesign

The pressing shortage of care professionals in the rather small municipality provided a suitable context to design innovative local primary care and community services. The current study has helped to shape the healthcare organisation in this community. Both during and following the project, a primary care centre was launched in line with community needs and some additional initiatives were set up to structurally embed equity and participation in the (health) policy of the community. This included training of healthcare professionals and volunteers on recognizing health illiteracy, which is considered an important predictor for health inequity [52], and using inclusive and clear written & oral health communication. All professionals and volunteers working in care and wellbeing in de local community were invited for the training activities, followed by a network event to strengthen links between different care professionals of the municipality. Additionally, the training provided access to useful materials and established connections to existing initiatives on health literacy. This approach aimed to equip healthcare professionals with the tools needed to improve patient understanding and engagement in managing their health and to reduce existing barriers in their organisations and services.

The local government also wanted to embed co-creation in a more structural way in their policy making. Therefore, citizen discussion platforms (“Dorpstafels”) were introduced. These are moments at which the local governments sits together with diverse citizens (including vulnerable groups) to discuss how the community can be further improved. The citizen discussion forum has for example already discussed findings of the activities of this study and has works on identifying ways to improve integrated primary care in the community. This has led to the opening of a new open house (KomAf) which is run mostly by volunteers to reduce barriers for vulnerable individuals to seek help with any question that they may have, including digital skills and access to computers. This open house aims to further extend integrated care and closely collaborates with the primary care centre.

### The added value of the living lab approach

Citizens can facilitate change in a range of health, care, and community services [7]. In line with Valentijn et al. [3] who emphasize equity, inter-sectoral collaboration, and political support as key factors for integrated primary care, the present study describes a process of co-creating a future-proof primary care strategy with an integrated open multidisciplinary ecosystem, putting the focus on prevention and close cooperation between different actors in the health and care sector. Complementing survey-based data collection with co-creation activities is highly valuable, as it promotes comprehension of the needs of the broad community and enables rich qualitative findings that provide actionable insights. However, such a study design also entails certain challenges. Achieving these results was only possible through the extensive, long-term commitment of both the living lab and the local government. The study design could have been further strengthened by including a larger sample size in the second survey study and by conducting a greater number of co-creation sessions to facilitate more extensive stakeholder consultation. Finally, it would have been valuable to include an impact assessment following the study activities and the resulting community initiatives, although impact measurement has proven challenging in living lab approaches [53].

Taken together, the current study shows that a living lab approach including a large stakeholder consultation and co-creation delivers relevant insights that facilitate the redesign of healthcare services in the community and can guide implementation efforts. Partnerships between living labs and local governments are promising in assessing community’s needs in terms of reorganisation of integrated primary healthcare and implementing local preventive initiatives supported by citizens and other stakeholders.

### Implications for policy and practice

Over the past years, an increase in research on the development of a health ecosystem approach for regional healthcare can be observed (e.g., [52]). Such an approach will lead to better care and will benefit citizens, health professionals and society. However, an important challenge for health promotion and healthcare proves to be health literacy, which is lower in vulnerable populations, e.g., elderly or lonely individuals and patients with (mental) health problems. This should be taken into account by policy makers and care professionals when designing high-quality integrated primary care in the community. The current work aims to share processes and results that can inspire further community consultation and healthcare reorganisation. The combination of large-scale surveys and tailored co-creation appears feasible to execute and allows to collect valuable information. Therefore, there might be opportunities to upscale and deploy the processes and results in larger health networks or even at a national level. Interesting avenues for future research are situated in extending the approach to localised implementation research into health promotion interventions, with special attention to the inclusion of residents in a situation of vulnerability. 

## Supplementary Information

Supplementary Material 1.

Supplementary Material 2.

## Data Availability

The datasets used and/or analysed during the current study are available from the corresponding author on reasonable request.

## Abbreviations

GP: General practitioner
HLS-Q12: European Health Literacy Questionnaire - short version
SF-RAND-36: Short Form Health Survey
AUDIT: Alcohol Use Disorder Identification Test
