# Supplementary material for: Living labs as catalysts for integrated primary care: insights from a comprehensive community survey and co-creative design of community-based prevention and primary care
Source: BMC Prim Care. 2026 Jul 8;27:346. doi: 10.1186/s12875-026-03467-3 (PMC13640197; doi:10.1186/s12875-026-03467-3)
Supplement: Supplementary file 1 [file 12875_2026_3467_MOESM1_ESM.docx]

Supplementary file 1

Supplementary Findings and materials of study 1: A survey study aimed to engage a sample that reflected the local diversity to provide insight into population health, health literacy and its predictors as well as user needs for primary healthcare and preventive initiatives.

# Supplementary results of study 1

Participants selected their top 5 of preferred disciplines for an interdisciplinary primary care centre out of a list of 13 care professions, besides the need for family doctors (Table S1). For the five most listed professions their mean rank was taken into account to determine an overall top 5. Adding a dentist to a primary care centre was considered most essential, followed by a nurse, psychologist, dietician and a social worker. Other disciplines that were proposed beyond the list of 13, were ophthalmologist, (*N* = 82), physiotherapist (*N* = 37), dermatologist (*N* = 23), gynecologist (*N* = 21), and osteopath (*N* = 20). Exploratory analyses showed only small age differences, e.g. a nurse being less important for younger individuals and a burn-out coach being more important for younger individuals.

Table S1.1. Preferred health professions for the primary care centre in addition to the general practitioner.

| **Care profession** | **N** | **Mean rank** |
| --- | --- | --- |
| Dentist | 869 | 1.31 |
| Nurse | 392 | 2.29 |
| Psychologist | 532 | 2.52 |
| Dietician | 263 | 3.17 |
| Social worker | 275 | 3.29 |
| Burn-out coach | 191 | 3.45 |
| Medical secretary | 184 | 2.64 |
| Occupational therapist | 182 | 3.38 |
| Speech therapist | 170 | 3.50 |
| Personal trainer (on referral) | 85 | 3.25 |
| Midwife | 82 | 3.33 |
| Sexologist | 37 | 3.81 |
| Smoking cessation counsellor | 19 | 3.32 |

*Note.* N is the number of times a particular profession is mentioned in the top 5. Lower mean rank indicates a higher need to include the profession in the care centre. Physiotherapists were not included in the list because this group was already well represented in the municipality.

# Supplementary materials of study 1: English translation of the questionnaire

## PART 1: Background information

*The following questions are about you or the person for whom you are filling in the survey.*

1. What is your date of birth? *Providing the* y*ear is sufficient*.

____________

2. What is your gender?
   - Male
   - Female
   - X
   - I'd rather not answer this question
3. What is your highest obtained degree?
   - No diploma or certificate
   - Primary education
   - Lower secondary education (13 to 15 years)
   - Upper secondary education (16 to 18 years)
   - Non-university higher education (HB05, graduate, professional bachelor, BanaBa) (18 years and older)
   - University Higher Education (Academic Bachelor, Master, ManaMa, Doctorate) (18 years and over)
   - Other, namely ...
4. How good is your knowledge of Dutch?
   - Mother tongue
   - Very good
   - Good
   - Rather limited
   - I have no knowledge of Dutch
5. How many people are currently part of your household, including yourself?
   - 1
   - 2
   - 3
   - 4
   - 5
   - More than 5
6. Which of the following categories best describes your employment situation?
   - Student
   - Full-time employment
   - Working part-time
   - Not employed: looking for work
   - Not employed: not looking for work (e.g., stay-at-home parent)
   - Not employed: long-term sick or (permanently) incapacitated for work
   - Retired
7. How do you make ends meet with your total household income per month? *Your total household income is the total budget that you have available as a family to pay for all expenses.*
   - Very easy
   - Easy
   - Neither easy nor difficult
   - Difficult
   - Very difficult

## PART 2: Health and well-being

*The following questions concern the theme of health and well-being.*

8. How important is health to you? Indicate on a scale from 0 (= not important at all) to 10 (very important). Circle the appropriate number.

| Not important at all | 0 | 1 | 2 | 3 | 4 | 5 | 6 | 7 | 8 | 9 | 10 | Very important |
| --- | --- | --- | --- | --- | --- | --- | --- | --- | --- | --- | --- | --- |

9. How do you rate your health right now?
   - Very good
   - Good
   - Average
   - Poor
   - Very poor
10. To what extent do you agree with the statements below?

|  | Certainly | Yes | More or less | No | Certainly not |
| --- | --- | --- | --- | --- | --- |
| I feel good about myself |  |  |  |  |  |
| I experience an emptiness around me |  |  |  |  |  |
| There are plenty of people I can fall back on in case of problems |  |  |  |  |  |
| I have a lot of people I can rely on completely |  |  |  |  |  |
| There are plenty of people I feel connected to |  |  |  |  |  |
| I miss people around me |  |  |  |  |  |
| I often feel abandoned |  |  |  |  |  |

11. Do you currently have a regular GP?
    - Yes
    - No (go to question 13)
12. In which municipality is your GP located?

*Only fill in this question if you have a regular GP.*

- Vorselaar
- Grobbendonk
- Herentals
- Lille
- Malle
- Zoersel
- Zandhoven
- Another municipality, namely ...

13. What factors do you consider important when choosing a GP?

*Rank the factors below by putting a number from 1 (most important factor) to 5 (least important factor) in front of them. Add a factor yourself if the list is incomplete for you.*

____ Distance to the GP

____ Being able to see the doctor quickly

____ GP recommended by friends or family

____ General practitioner is part of a group practice

____ Another factor, namely __________________________________

## PART 3: Primary care practice PIOEN

*The following questions are about the new primary care centre PIOEN in Vorselaar.*

14. A general practitioner will start working in the PIOEN health home in Vorselaar from 1 October. Which other (care) professions do you think are important to add *in the long term*?

*Choose a maximum of 5.*

- Dentist
- Medical reception/practice assistant
- Nurse
- Tobaccoologist
- Dietitian
- Personal trainer/BOV coach (exercise on referral)
- Psychologist
- Speech therapist
- Occupational therapist
- Social worker
- Midwife
- Sexologist
- Burn-out coach

15. Sort your chosen professions from question 14 according to importance. Put the profession that you think is most important in place 1.
16. Are there any (care) professions that are not covered in question 14 but that you would like to find in PIOEN?

___________________________________________________________________

___________________________________________________________________

___________________________________________________________________

17. Do you have additional ideas or comments about the new primary care centre PIOEN?

___________________________________________________________________

___________________________________________________________________

___________________________________________________________________

## PART 4: Health literacy

*The following questions are about receiving, understanding, assessing and applying information about health.*

18. Below are some statements. Indicate on the scale (from very difficult to very easy) **how easy it is for you to...**

|  | Very difficult | Difficult | Easy | Very easy |
| --- | --- | --- | --- | --- |
| … Find information about treatments or diseases that affect you |  |  |  |  |
| … understand what to do during a medical emergency |  |  |  |  |
| … Assess the pros and cons of different treatment options |  |  |  |  |
| … follow the instructions regarding your medication |  |  |  |  |
| … Find information on how to manage mental health issues such as stress or depression |  |  |  |  |
| … understand why screening tests are important (e.g. for breast and colon cancer, blood pressure measurement, blood sugar test) |  |  |  |  |
| … assess which information on health risks in the media is reliable |  |  |  |  |
| … decide how to protect yourself from illness after advice from family and friends |  |  |  |  |
| … Find information about healthy activities such as exercise and healthy nutrition |  |  |  |  |
| … Understand information on food packaging |  |  |  |  |
| … assess what behaviours in daily life are related to your health |  |  |  |  |
| … Make decisions to improve your health |  |  |  |  |
| … to understand your GP |  |  |  |  |

19. To what extent do you agree with the statement below? Indicate on a scale from 0 (= strongly disagree) to 10 (completely agree). Circle the appropriate number.

*I have at least one caregiver by whom I feel understood and supported.*

| Strongly disagree | 0 | 1 | 2 | 3 | 4 | 5 | 6 | 7 | 8 | 9 | 10 | Totally agree |
| --- | --- | --- | --- | --- | --- | --- | --- | --- | --- | --- | --- | --- |

## PART 5: Health prevention

*The following questions are about health prevention. The goal of prevention is to prevent diseases or complications for health problems by promoting and protecting your own health.*

20. How important is a healthy lifestyle to you? Indicate on a scale from 0 (= not important at all) to 10 (very important). Circle the appropriate number.

| Not important at all | 0 | 1 | 2 | 3 | 4 | 5 | 6 | 7 | 8 | 9 | 10 | Very important |
| --- | --- | --- | --- | --- | --- | --- | --- | --- | --- | --- | --- | --- |

21. What factors do you think have the most influence on health?

*Rank the factors below by putting a number from 1 (most important factor) to 5 (least important factor) in front of them. Add a factor yourself if the list is incomplete for you.*

____ Healthy Diet

____Physical exercise

____Alcohol consumption

____Smoking

____ Mental health

____Sleep

____ Other, namely _________________

22. To what extent are you willing to use a health tool, such as a website or app, to measure your health and take preventive actions in the field of health? Indicate on a scale from 0 (= not at all) to 10 (definitely). Circle the appropriate number.

| Not at all | 0 | 1 | 2 | 3 | 4 | 5 | 6 | 7 | 8 | 9 | 10 | Certainly |
| --- | --- | --- | --- | --- | --- | --- | --- | --- | --- | --- | --- | --- |

23. To what extent do you think it is the task of the municipality to work on health prevention? Indicate on a scale from 0 (= not at all) to 10 (definitely). Circle the appropriate response.

| Not at all | 0 | 1 | 2 | 3 | 4 | 5 | 6 | 7 | 8 | 9 | 10 | Certainly |
| --- | --- | --- | --- | --- | --- | --- | --- | --- | --- | --- | --- | --- |

24. How can Vorselaar better support residents in terms of health prevention?

___________________________________________________________________

___________________________________________________________________

___________________________________________________________________
