# Supplementary material for: Living labs as catalysts for integrated primary care: insights from a comprehensive community survey and co-creative design of community-based prevention and primary care
Source: BMC Prim Care. 2026 Jul 8;27:346. doi: 10.1186/s12875-026-03467-3 (PMC13640197; doi:10.1186/s12875-026-03467-3)
Supplement: Supplementary file 2 [file 12875_2026_3467_MOESM2_ESM.docx]

Supplementary file 2

Supplementary Findings and materials of study 2: study 2 provides an overview of different activities and actions to support the quality of integrated primary care in the community, including a primary care centre and its activities.

# Supplementary results of study 2

The demographics of survey participants can be consulted in table S2.

Table S2.1. Demografics of participants in the survey on health, lifestyle and prevention

|  | **Frequency** | **Percentage** |
| --- | --- | --- |
| **Gender** | **281** |  |
| Male | 95 | 33.81 |
| Female | 186 | 66.19 |
| X | 0 | 0.00 |
| Prefer not to say | 0 | 0.00 |
| **Age category** | **280** |  |
| 18-39 years old | 48 | 17.14 |
| 40-59 years old | 88 | 31.43 |
| 60-79 years old | 135 | 48.21 |
| >80 years old | 9 | 3.21 |
| **Highest education degree** | **281** |  |
| No degree | 10 | 3.56 |
| Primary education | 14 | 4.98 |
| Lower secondary education | 98 | 34.88 |
| Higher secondary education | 109 | 38.79 |
| Non-university higher education | 46 | 16.37 |
| University education | 4 | 1.42 |
| **Professional situation** | **281** |  |
| Student, apprentice | 4 | 1.42 |
| Working fulltime | 100 | 35.59 |
| Working parttime | 35 | 12.46 |
| Not employed - looking for work | 6 | 2.14 |
| Not employed - not looking for work | 9 | 3.20 |
| Not employed - long-term illness or permanently incapacitated for work | 9 | 3.20 |
| Retired | 114 | 40.57 |
| Other | 9 | 3.20 |
| **Getting by on family income *(subjective income)*** | **281** |  |
| Very easy | 47 | 16.73 |
| Easy | 99 | 35.23 |
| Neither easy nor difficult | 116 | 41.28 |
| Difficult | 19 | 6.76 |
| Very difficult | 5 | 1.78 |
| **Living situation** | **281** |  |
| Living alone | 42 | 14.95 |
| Living alone with children | 18 | 6.41 |
| Living together with partner without children | 121 | 43.06 |
| Living together with partner and children | 91 | 32.38 |
| Living with family | 11 | 3.91 |
| Living with friends | 0 | 0.00 |
| Other | 3 | 1.07 |

***Interest in prevention communication and activities***

The results of the survey in study 2 indicate that over half of participants (*N* = 148) did not read communications on health prevention from the municipality nor participated in activities organized. The most popular articles in the current participant group were on physical exercise and fall prevention (*N* = 91), cancer screening (*N* = 76) and air quality (*N* = 69). In terms of activities, a weekly hike or bike ride (*N* = 20), Tournée Minéral (a Flemish initiative to reduce alcohol consumption) (*N* = 20) and activities on a public exercise bench guided by a physiotherapist (*N* = 19) were most attended in the current population. Participants could indicate which health topics they were interested in based on an existing list or by suggesting certain topics. The top ranked themes were diet, physical activity, sleep, mental health, dealing with stress, cancer screening and prevention, first aid, social health, work-life balance, and self-care (Table S2). However, only 5.1% (N = 14) of participants would very likely participate in health or prevention initiatives from the local government, local associations, or the primary care centre in the future. About 40.9% (*N* = 112) participants indicated that they would potentially participate, which means that most participants would rather not (48.2%; *N* = 132 ) or not at all (5.8%; *N* = 16) wish to participate in the future. Information about upcoming preventive initiatives should be communicated through the monthly municipal magazine (available online and on paper) (*N* = 214), flyers in the letterbox (*N* = 115), social media (*N* = 92), the multidisciplinary primary care centre and its website (*N* = 71), the municipality website (*N* = 69), the family doctor (*N* = 54), the town hall (*N* = 26), the library (*N* = 19), or other (*N* = 19). Finally, participants received some questions about a specific online accessible prevention tool, called BIBOPP (see also training section). Most respondents (73.1%, *N* = 196) had never heard of BIBOPP, 19.4% (*N* = 52) said they had heard of BIBOPP, and 7.5% (*N* = 20) were not quite sure. Thirty-five people (13.1%) had already used the application and about 1 in 3 said they plan to use BIBOPP in the future (32.8%; N = 88). An additional third of participants were still unsure whether to use it (*N* = 88) and fifty-seven residents (21.3%) said they do not want to use the digital tool BIBOPP.

*Table S2.2. Health themes of interest to the participants of the survey in study 2.*

| **Theme** | **N** | **Theme (continued)** | **N** |
| --- | --- | --- | --- |
| Diet | 175 | Illness: cardiovascular disease | 50 |
| Exercise | 164 | Illness: dementia | 48 |
| Sleep | 145 | Oral health | 43 |
| Mental health | 115 | Loneliness | 32 |
| Dealing with stress | 100 | Illness: diabetes | 29 |
| Cancer screening and prevention | 92 | Fall prevention | 26 |
| First aid | 87 | Living quality | 24 |
| Social health | 74 | Medication use | 23 |
| Work-life balance | 65 | Alcohol use | 20 |
| Selfcare | 59 | Illness: other (self-specified) | 10 |
| Environment (e.g. air quality) | 57 | Use of other drugs or addiction | 3 |
| Children’s health | 54 | Other (self-specified) | 2 |

# Supplementary materials of study 2: English translation of the questionnaire

## PART 1: Background information

*The following questions are about you or the person for whom you are filling in the survey.*

1. What is your year of birth? *E.g. 1990*.

____________

2. What is your gender?
   - Male
   - Female
   - Other, namely ______________________________
   - I'd rather not answer this question
3. What is your highest obtained degree?
   - No diploma or certificate
   - Primary education
   - Primary or upper secondary education
   - Non-university higher education (HB05, graduate, professional bachelor, BanaBa) (18 years and older)
   - University Higher Education (Academic Bachelor, Master, ManaMa, Doctorate) (18 years and over)
   - Other, namely ______________________________________
4. Which of the following categories best describes your employment situation?
   - Student, apprentice or in vocational training
   - Full-time employment
   - Working part-time
   - Not employed: looking for work
   - Not employed: not looking for work (e.g. stay-at-home parent)
   - Not employed: long-term sick or (permanently) incapacitated for work
   - Retired
   - Other, namely ______________________________________
5. How do you make ends meet with your total household income per month? *Your total household income is the total budget that you have available as a family to pay for all expenses.*
   - Very easy
   - Easy
   - Neither easy nor difficult
   - Difficult
   - Very difficult
6. What is your current living situation?
   - Living alone
   - Living alone with children
   - Living with partner without children
   - Living with partner and child(ren)
   - Living with family, such as parent(s) or child(ren)
   - Living with friends
   - Other, namely __________________________________

## PART 2: Health and well-being

*The following questions concern the theme of health and well-being.*

7. What do you think about your **physical/bodily** health in general? (E.g. How fit do you feel?)
   - Very good
   - Good
   - Average
   - Bad
   - Very bad
8. Compared to a year ago, how would you rate your **physical/bodily** health in general now? (E.g. How fit do you feel?)
   - Much better than a year ago
   - Slightly better than a year ago
   - About the same as a year ago
   - Slightly worse than a year ago
   - Much worse than a year ago
9. What do you think about your **mental/psychological health** in general? (E.g. How good do you feel about yourself?)
   - Very good
   - Good
   - Average
   - Bad
   - Very bad
10. Compared to a year ago, how would you rate your **mental/psychological** health in general now? (E.g. How good do you feel about yourself?)
    - Much better than a year ago
    - Slightly better than a year ago
    - About the same as a year ago
    - Slightly worse than a year ago
    - Much worse than a year ago
11. What do you think about your **social** health in general? (E.g. social contacts with others and participation in community activities)?
    - Very good
    - Good
    - Average
    - Bad
    - Very bad
12. Compared to a year ago, how would you rate your **social** health in general now)? (E.g., socializing with others and participating in community activities)
    - Much better than a year ago
    - Slightly better than a year ago
    - About the same as a year ago
    - Slightly worse than a year ago
    - Much worse than a year ago
13. Do you have a long-term illness or chronic condition (health problem, longer than 3 months)?
    - Yes
    - No
14. *Only to be filled in if you* ***answered*** *'yes'* ***to question 13****:* To what extent does this health problem affect your daily life? *Circle the number that applies to you.*

| Not at all | 0 | 1 | 2 | 3 | 4 | 5 |  | 6 | 7 | 8 | 9 | 10 | Very much |
| --- | --- | --- | --- | --- | --- | --- | --- | --- | --- | --- | --- | --- | --- |

15. To what extent are you able to carry out your daily activities such as walking, going up stairs, carrying groceries, moving a chair?
    - Totally
    - Mostly
    - Somewhat
    - A little bit
    - Not at all

## PART 3: Lifestyle

*The following questions are about your lifestyle and behavior in the context of health. Various themes that are discussed are nutrition, exercise, sleep, alcohol use and tobacco use.*

**Nutrition**

16. How often do you eat fruit*? Drinking (fresh) fruit juice should NOT be counted.*
    - Daily
    - 4-6 times per week
    - 1-3 times per week
    - Less than 1 time per week
    - Never
17. How often do you eat vegetables? *Eating potatoes or drinking vegetable juice should NOT be included.*
    - Daily
    - 4-6 times per week
    - 1-3 times per week
    - Less than 1 time per week
    - Never

**Physical exercise**

18. How often do you exercise for at least half an hour on top of your daily activities? (e.g. cycling, brisk walking, swimming, gardening, etc.)?
    - Daily
    - 4-6 times per week
    - 1-3 times per week
    - Less than 1 time per week
    - Never

**Sleep**

*To what extent do the statements below apply to you?*

19. I find it difficult to fall asleep in bed.
    - Rarely or never
    - Sometimes
    - Often
    - (Almost) always
20. While sleeping, I regularly wake up at night.
    - Rarely or never
    - Sometimes
    - Often
    - (Almost) always
21. I snore when I sleep.
    - I don't know
    - Rarely or never
    - Sometimes
    - Often
    - (Almost) always
22. I feel excessively tired during the day.
    - Rarely or never
    - Sometimes
    - Often
    - (Almost) always

**Alcohol consumption**

23. Do you use alcoholic beverages? (beer, wine, spirits)
    - Yes
    - No
24. *Only to be filled in if you* ***answered*** *'yes'* ***to question 23:*** How often do you drink alcohol?
    - 4 or more times per week
    - 2 to 3 times per week
    - 2 to 4 times per month
    - Monthly or less
    - Never
25. *Only to be filled in if you* ***answered*** *'yes'* ***to question 23:*** When you drink, how many standard glasses (*) do you usually drink in a day?
    - 10 or more
    - 7-9 glasses per day
    - 5 of 6
    - 3 of 4
    - 1 of 2

*A standard glass of alcohol corresponds to a 250 ml glass of beer with 5% alcohol, 100 ml of wine with 12% alcohol, 40 ml of liqueur (e.g. gin) with 25% alcohol, 30 ml of spirits (e.g. brandy) with 40% alcohol.

26. *Only to be filled in if you* ***answered*** *'yes'* ***to question 23:*** How often does it happen that you drink six or more standard units (*) on a single occasion?
    - Daily or almost daily
    - Weekly
    - Monthly
    - Less than monthly
    - Never

**Tobacco consumption**

27. Do you smoke? *Several answers are possible*.
    - No, I have never smoked
    - No, I used to smoke
    - Yes, I smoke regular cigarettes, cigarillos or cigars
    - Yes, I smoke e-cigarettes with nicotine
    - Yes, I smoke e-cigarettes without nicotine
28. *Only to be filled in if you* ***answered*** *'yes, ...'* ***to question 27:*** Are you thinking of quitting smoking?
    - Yes
    - No

## PART 4: Health prevention

*The following questions are about health prevention. The goal of prevention is to prevent diseases or complications for health problems by promoting and protecting your own health.*

29. *Only to be completed by* ***women aged 25 to 64****:* Was your last Pap smear more than 3 years ago?
    - Yes
    - No
    - I don't know
30. *Only to be completed by* ***women aged 50 to 69****:* Was your last breast cancer screening more than 2 years ago?
    - Yes
    - No
    - I don't know
31. *To be completed only by* ***people between 50 and 74 years of age****:* Was your last colon cancer examination more than 2 years ago?
    - Yes
    - No
    - I don't know
32. The local government regularly organises and supports activities related to health prevention.

Several articles on this theme have already appeared in **InfoVorselaar, on social media and on the website of primary care centre PIOEN** (pioenvorselaar.be/blijfgezond). Which article(s) have you read? *Several answers are possible.*

- None of these
- InfoVorselaar article January about **cancer screening**
- Article InfoVorselaar February about **BIBOPP (tailor-made health plan)**
- Article InfoVorselaar March about **oral health**
- Article InfoVorselaar April about **exercise and fall prevention**
- Article InfoVorselaar May on **ventilation and air quality**
- Social media articles **about** __________________________________
- Other, namely _________________________________

33. Did you already take a look at the website **pioenvorselaar.be/blijfgezond**?
    - Yes
    - No
    - I don't know
34. Which activity(ies) did you participate in? *Several answers are possible.*
    - None of these
    - Exercise bench (“Beweegbank”)
    - Sports activities during the Easter holidays
    - Weekly walks or bike rides
    - Theatre about dementia
    - (Information session) smoking cessation course
    - Tournée Minérale (not drinking alcohol for a whole month)
    - Workshop cheap healthy cooking
    - Facebook tips on air quality/ventilation
    - Other, namely ___________________________________
35. What are the chances that you will participate in such or other health or prevention initiatives from the local government, local associations, health insurance funds or PIOEN in the future?
    - Very large
    - Probably yes
    - Probably not
    - Not at all
36. What topics do you care about in terms of health prevention? *Several answers are possible.*
    - Nutrition
    - Physical exercise
    - Sleep
    - Social health (social contacts and participation in society)
    - Mental/Psychological Health
    - Dealing with stress
    - Work-life balance
    - Oral health
    - Alcohol consumption
    - Tobacco consumption
    - Medication use
    - Use of other (stimulant) substances/addiction
    - Children's health
    - Cancer screening/cancer prevention
    - Fall prevention
    - Disease: information on diabetes
    - Disease: information on dementia
    - Disease: information on cardiovascular disease
    - Disease: information on another disease, namely_________________
    - First aid
    - Self-care
    - Loneliness
    - Quality of living (how good or bad is your living situation)
    - Environment, space and environment (e.g. air quality)
    - Others, namely_____________________________________
37. What helps you to participate in a health prevention activity? (e.g. free, nearby, in group, child-friendly, in the evening or during the day, not too difficult, ...)

___________________________________________________________________

___________________________________________________________________

___________________________________________________________________

38. Through which channels are you preferably informed of such activities? *Several answers are possible.*
    - General practitioner
    - (Website) primary care centre PIOEN
    - Town hall
    - Library
    - Info Vorselaar (municipality publication)
    - Website Vorselaar
    - Social media such as facebook, instagram, ...
    - Flyer in the mailbox
    - Social services
    - 't Kiertje
    - Other, namely ..........................................................................................
39. The municipality offers the **free** website BIBOPP. With BIBOPP you can create a personal action plan about your health. It helps you to lower your risk of disease. You fill in the health guide, receive immediate tailor-made advice and an action plan with activities in your area. You can share your results directly with your GP if you wish.

Have you heard of the free online platform BIBOPP in Vorselaar before?

- Yes
- No
- I don't know

Have you already completed the BIBOPP questionnaire (health guide)?

- Yes
- No, I intend to
- No, I don't intend to
- I don't know

40. With the help of ItsMe®, you can log in to various services, ranging from your bank's website, to telecom and government services to insurers. Are you already using ItsMe®?
    - Yes
    - No
    - I don't know
41. Do you have any additional ideas for your local government in the field of health prevention?

___________________________________________________________________

___________________________________________________________________

___________________________________________________________________
